# Supplementary material for: Changes in Soil Aggregate Carbon Components and Responses to Plant Input during Vegetation Restoration in the Loess Plateau, China
Source: Plants (Basel). 2024 Sep 2;13(17):2455. doi: 10.3390/plants13172455 (PMC11396830; doi:10.3390/plants13172455)
Supplement: Supplementary file 1 [file plants-13-02455-s001.zip › plants-3173683-supplementary.pdf]

Supplementary Information

1. Vegetation survey

Five 1m×1m vegetation survey areas were set up in each sampling area using a five-point sampling method, and the name, quantity, height, and coverage of each plant species in the survey area were recorded, as well as the individual number and total number of all species in the quadrate. The height of the plant was measured with a meter ruler, and the coverage was estimated by visual measurement [8]. In addition, three samples were randomly selected to collect the above-ground biomass of understory vegetation by drying to constant weight and recording its weight. All survey work was performed by two investigators to reduce human error, and all survey data were compiled for the subsequent analysis.

2. Sample collection

Soil sample collection: We dug a flat section with a length, width, and height of 1m in the standard sampling area. We used undisturbed soil in the three soil layers of 0-20cm, 20-40cm, and 40-60cm of each section with aluminum boxes. We sealed the aluminum boxes and placed them in an incubator equipped with ice boxes and ice packs and brought them back to the laboratory. After air drying at room temperature, it was broken into about 8mm soil blocks along the natural fracture surface of the soil for the separation and determination of soil aggregates. In each sample plot, nine sample points were chosen using a soil drill with a 9 cm diameter in accordance with the "S" type. Following the removal of stones and plant residues, soil samples from three soil layers were collected and mixed well with samples from the same soil layer. The samples were returned to the laboratory after being screened for 2 mm. The quarter approach was used to split the collected soil samples into two sections. Part of the samples were stored in a refrigerator at 4℃ for the determination of microbial biomass, and the rest of the soil was naturally air-dried for the determination of soil physical and chemical characteristics.

Collection of plant residues: A 1m×1m plant residue collection area was set up in each standard sampling area; litter residues of trees, shrubs, and herbs were collected; and root residues of 0-20cm, 20-40cm, and 40-60cm soil layers were dug into envelopes and brought back to the laboratory for drying at 60℃ to determine plant properties.

3. Determination of soil organic carbon structure

A Fourier infrared spectrometer (NicoletIS50, USA) was used to dry the soil sample at 60℃ for 16h to constant weight, while the KBr (spectrally pure) was dried at 120℃ for 4h. After drying, the soil sample and KBr (spectrally pure) were fully ground in an agate mortar at the ratio of 1:200 to ensure that the sample and KBr were mixed well. The mixed sample was transferred to the tablet pressing device and evenly filled. The tablet was pressed for 30s at 20MPa and the thickness of the tablet was controlled to about 1mm. The pressed sample was measured on the infrared transmission platform of the Fourier transform infrared spectrometer and scanned 32 times with a resolution of 4cm^{-1}. Background acquisition was required before measurement, and the atmospheric background was automatically deducted during scanning. In this study, the main characteristic peaks of typical functional groups of the absorption peaks of soil samples are 3455, 2928, 2510, 1622, 1434, and 1035cm^{-1}. Additionally, 3455cm^{-1} is the phenolic compound -OH stretching vibration from soil carbohydrates, 2928cm-1 is the C-H stretching vibration from the aliphatic group, 2510cm-1 is the -OH stretching vibration of carboxyl group C, 1622cm^{-1} is the C=O of aromatic C. The absorption peak at 1434cm^{-1} is the deformation vibration of methylene CH_2, and the absorption peak at 1035cm^{-1} is the C-O vibration in polysaccharides. It was found that the organic compounds in the farmland and robinia acacia forest were mainly phenolic OH, aliphatic CH, aromatic C=O, polysaccharide C-O, etc.

4. Determination of soil physical and chemical properties and organic carbonation structure

Soil organic carbon (soil organic carbon) was determined by potassium dichromate oxidation and an external heating method, and soil total nitrogen (TN) was determined by the Kjeldahl method. Soil nitrate nitrogen ($NO3^{-}N$) and ammonium nitrogen ($NH4^{+}N$) were determined by the potassium chloride extraction method. Soil particulate organic carbon and mineral-bound organic carbon were determined by the extraction method with a sodium hexametaphosphate solution. Plant organic carbon (OC) was determined using the potassium dichromate external heating method. The plant total nitrogen (TN) and total phosphorus (TP) were boiled by the H_2SO_4-H_2O_2 method. Plant lignin, cellulose, and washing fiber were determined by boiling and sulfuric acid hydrolysis. For the specific method, refer to Soil Agrochemical Analysis Guide 2020. The Fourier transform infrared spectrometer was used to determine the soil organic carbon structure. Polysaccharide carbon and methylene carbon were chosen as active carbon components, while aromatic, aliphatic, carboxyl, and phenolic chemicals were chosen as non-active carbon components. By integrating the wavelength of the corresponding absorbance peak, one can obtain the entire area beneath the characteristic peak. We determined the ratio, or relative peak area, of activated carbon (AC) to inactivated carbon (IC).

Table S1. Stability of soil aggregates in *Robinia pseudoacacia* forest with different years after returning farmland. Note:Lowercase letters a,b,c,d indicate significant differences between plots of different afforestation years (p<0.05) .

Item	Soil layer (cm)	FL	RP16	RP22	RP32	RP47
Mean weight diameter (MWD)	0-20	1.32±0.08a	0.92±0.15b	0.75±0.12b	0.63±0.09b	0.90±0.14b
	20-40	0.70±0.12a	0.75±0.14a	0.75±0.09a	0.72±0.11a	0.66±0.15a
	40-60	0.50±0.14a	0.61±0.12a	0.53±0.12a	0.79±0.2a	0.45±0.03a
Geometric mean diameter (GMD)	0-20	0.77±0.08a	0.26±0.08b	0.35±0.08b	0.42±0.04b	0.24±0.05b
	20-40	0.28±0.05a	0.36±0.1a	0.36±0.07a	0.35±0.07a	0.41±0.12a
	40-60	0.72±0b	0.48±0.12ab	0.51±0.1ab	0.33±0.13ab	0.61±0.05a
> 0.25mm water-stable aggregate content	0-20	0.84±0.02a	0.72±0.1ab	0.67±0.11ab	0.56±0.08b	0.69±0.04ab
	20-40	0.64±0.03a	0.66±0.12a	0.66±0.09a	0.64±0.1a	0.58±0.14a
	40-60	0.49±0.1ab	0.53±0.1ab	0.47±0.11ab	0.74±0.09a	0.39±0.02b
> 0.25mm mechanically stable aggregate content	0-20	0.98±0a	0.93±0.02ab	0.92±0.04ab	0.89±0.03b	0.92±0.01ab
	20-40	0.97±0.01a	0.94±0.01a	0.93±0.01a	0.92±0.02a	0.93±0.03a
	40-60	0.95±0.01a	0.93±0.01a	0.92±0.02a	0.93±0.01a	0.93±0.01a

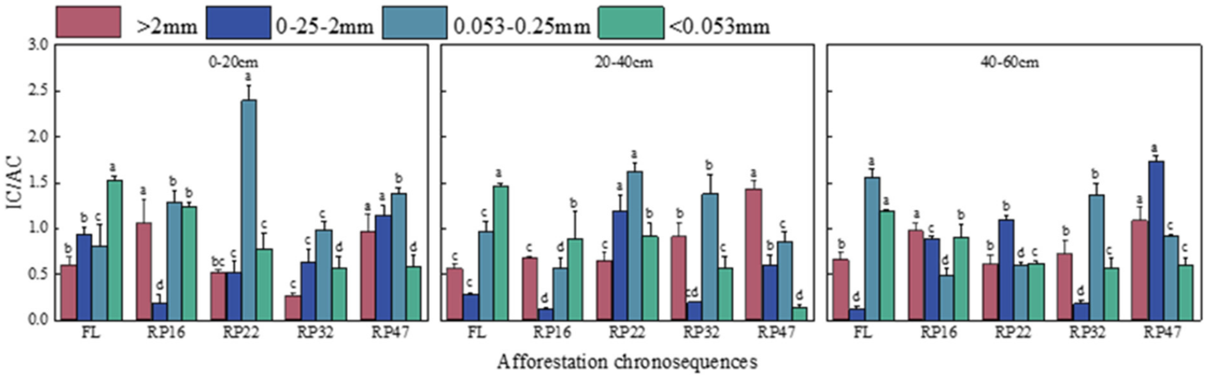

Figure S1. The ratio difference between the mass fraction of non-active carbon and active carbon of soil organic carbon in different grain grades of the *Robinia pseudoacacia* forest in different years. Note:Lowercase letters a,b,c,d indicate significant differences between plots of different afforestation years (p<0.05) .

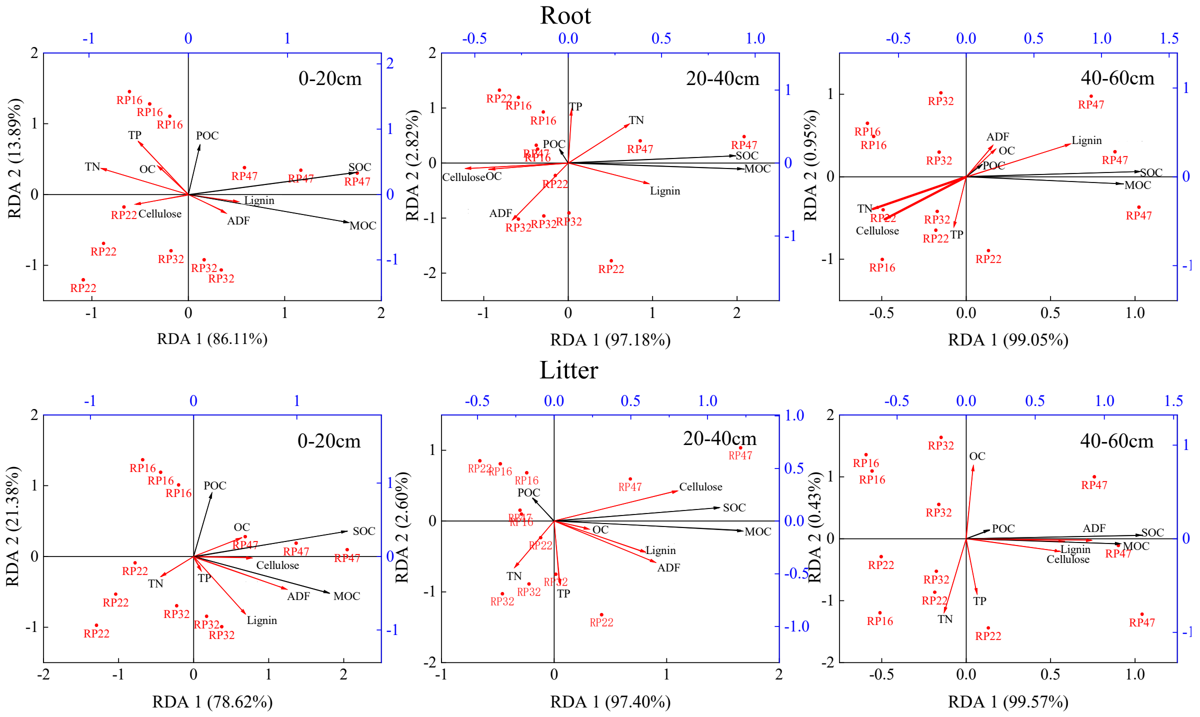

Figure S2. Redundancy analysis of organic carbon component litter and root input in soil aggregates of *Robinia pseudoacacia* of different years.
